# CT Coronary Angiography: Technical Approach and Atherosclerotic Plaque Characterization

**DOI:** 10.3390/jcm12247615

**Published:** 2023-12-11

**Authors:** Serena Dell’Aversana, Raffaele Ascione, Raffaella Antonia Vitale, Fabrizia Cavaliere, Piercarmine Porcaro, Luigi Basile, Giovanni Napolitano, Marco Boccalatte, Gerolamo Sibilio, Giovanni Esposito, Anna Franzone, Giuseppe Di Costanzo, Giuseppe Muscogiuri, Sandro Sironi, Renato Cuocolo, Enrico Cavaglià, Andrea Ponsiglione, Massimo Imbriaco

**Affiliations:** 1 Department of Radiology, Santa Maria Delle Grazie Hospital, ASL Napoli 2 Nord, 80078 Pozzuoli, Italy; dellaversanaserena@gmail.com (S.D.); giuseppe.dicostanzo@aslnapoli2nord.it (G.D.C.); enrico.cavaglia@asinapoli2nord.it (E.C.); 2 Department of Advanced Biomedical Sciences, University of Naples Federico II, 80131 Naples, Italy; raffoascio@gmail.com (R.A.); raffaellaantonia.vitale@unina.it (R.A.V.); fabr.cavaliere@studenti.unina.it (F.C.); piercarmine.porcaro@unina.it (P.P.); basileluigi92@gmail.com (L.B.); giovanni.esposito2@unina.it (G.E.); anna.franzone@unina.it (A.F.); massimo.imbriaco@unina.it (M.I.); 3 Cardiology Unit, San Giuliano Hospital, 80014 Naples, Italy; giovanni.napolitano@aslnapoli2nord.it; 4 Coronary Care Unit, Santa Maria delle Grazie Hospital, ASL Napoli 2 Nord, 80078 Pozzuoli, Italy; marco.boccalatte@aslnapoli2nord.it (M.B.); gerolamo.sibilio@aslnapoli2nord.it (G.S.); 5 Department of Radiology, ASST Papa Giovanni XXIII Hospital, Piazza OMS 1, 24127 Bergamo, Italy; g.muscogiuri@gmail.com (G.M.); sandro.sironi@unimib.it (S.S.); 6 School of Medicine and Surgery, University of Milano Bicocca, 20126 Milan, Italy; 7 Department of Medicine, Surgery and Dentistry, University of Salerno, 84081 Baronissi, Italy; rcuocolo@unisa.it

**Keywords:** CCTA, atherosclerosis, prognosis, radiomics

## Abstract

Coronary computed tomography angiography (CCTA) currently represents a robust imaging technique for the detection, quantification and characterization of coronary atherosclerosis. However, CCTA remains a challenging task requiring both high spatial and temporal resolution to provide motion-free images of the coronary arteries. Several CCTA features, such as low attenuation, positive remodeling, spotty calcification, napkin-ring and high pericoronary fat attenuation index have been proved as associated to high-risk plaques. This review aims to explore the role of CCTA in the characterization of high-risk atherosclerotic plaque and the recent advancements in CCTA technologies with a focus on radiomics plaque analysis.

## 1. Introduction

Nowadays, despite the incontrovertible progress both in the diagnostic and therapeutic field, atherosclerotic coronary disease remains the leading cause of death in European countries [1]. The primary cause of the disease consists in the formation of an atherosclerotic plaque in the intima of the coronary arteries. However, most coronary atherosclerotic plaques do not induce any symptoms; some can clinically manifest in numerous disease subtypes, generally classified as chronic coronary syndrome and acute coronary syndrome (ACS). Several pivotal papers and surveys, such as the Multi-Ethnic Study of Atherosclerosis (MESA), the Framingham Heart Study (FHS) and the Cardiovascular Health Study (CHS) showed that the prevalence of coronary artery disease (CAD) considerably increases with age and that men are affected more than women [2,3,4].

Nowadays, cardiac imaging includes multiple methods to diagnose CAD, both via the detection of the ischemic myocardium triggering the ischemic process or via the direct visualization of coronary artery stenoses [5]. 

Since most coronary stenoses do not cause any ischemia, generally the first approach in these patients is to identify myocardial ischemia by an imaging-based stress test, such as SPECT, PET or stress echocardiography and stress MRI. These functional non-invasive imaging methods are capable of detecting myocardial ischemia through ECG changes, wall motion abnormalities by stress CMR or stress echocardiography or perfusion changes by SPECT [6,7].

The second option consists of the direct visualization of the coronary anatomy, which can be obtained by invasive coronary angiography (ICA), which is the diagnostic reference standard in stable chronic coronary disease and acute coronary syndrome. This technique can also provide the measurement of the fractional flow reserve (FFR) to evaluate the stenosis significance [8]. 

However, ICA, besides being invasive, is a luminography technique, thus unable to provide any information about the vessel wall. New imaging modalities have been recently proposed for the non-invasive assessment of coronary artery disease. In particular, intravascular ultrasound (IVUS) and optical coherence tomography (OCT) can be valuable [9] while coronary computed tomography angiography (CCTA) has been established as an accurate and robust imaging technique which allows the quantification and characterization of coronary atherosclerosis [10,11]. Thanks to its high negative predictive value, CCTA is often used to conclusively exclude CAD in patients with a low pretest probability of disease. 

In acute coronary syndrome (ACS) [12], non-invasive cardiovascular imaging has a limited role. Electrocardiography is always the first test performed for STEMI and if positive, patients are immediately led to a coronary angiography room. In the case of NSTEMI, electrocardiographic findings together with troponin and echocardiography can be used to exclude other differential diagnoses in order to take a decision about invasive angiography [13]. 

Currently, CCTA has a critical role in ruling out CAD in patients with stable chest pain [14]. However, it cannot exclude other frequent causes of chest pain, such as acute myocarditis, myocardial infarction with normal coronary arteries and cardiomyopathies; for these reasons, researchers suggested the addition of a delayed scan to enhance myocardial scars via late iodine enhancement (LIE) and to evaluate extracellular volume (ECV) [15,16]. Furthermore, FFR derived from CCTA has emerged in the last decade as a promising tool for evaluation of the physiologic significance of a coronary artery stenosis [17].

Cardiac MRI has a limited role in the emergency setting, but it has a pivotal role early after coronary angiography, to determine risk stratification and prognosis and to detect complications [13,18].

In this review, we explore the role of CCTA in the characterization of high-risk atherosclerotic plaque and the recent advancements in CCTA technologies with a focus on radiomics plaque analysis.

## 2. Atherosclerosis

**Figure 1:**
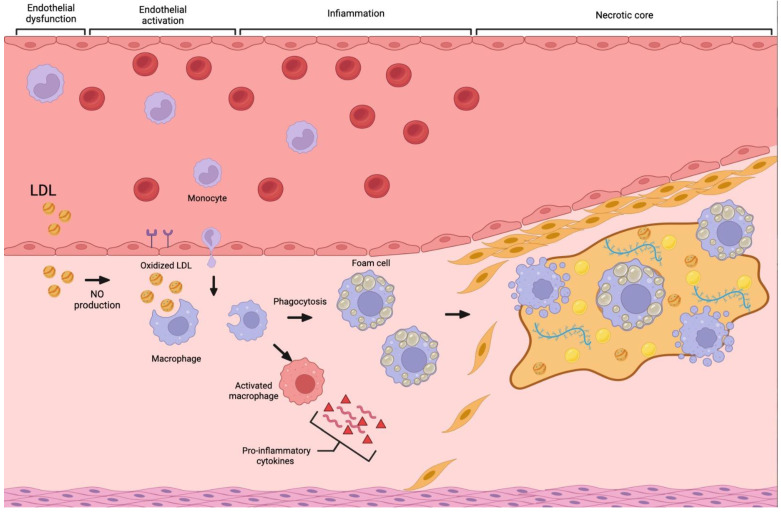
Exemplificative image of the atherosclerotic process.

Atherosclerosis is a slow chronic inflammatory disease characterized by the formation of lipid-rich plaques. This pathological process probably starts early in life as diffuse intimal thickening of the arterial walls, as the consequence of the deposition of lipids, fibrous tissue, calcium and smooth muscle cells in the intimal layer of coronary artery walls [5]. Furthermore, atherosclerotic risk factors such as hypertension, diabetes mellitus and smoke can damage the arterial wall, mainly at the endothelial level. Here, blood monocytes migrate to the subendothelial space and differentiate into macrophages that can incorporate low-density lipoproteins (LDL), becoming foam cells. These cells represent an essential element to the formation of fatty streaks that will progress to the atherosclerotic lesions [19,20,21]. The advanced stages of atherosclerotic lesions are represented by atheroma, fibro-lipid plaque and fibroatheroma which typically form in proximal coronary sectors and near bifurcations [10]. These plaques present a lipid or necrotic nucleus covered by a thick fibrous cap consisting of smooth muscle cells, collagen matrix and calcifications. Those with a thinner fibrous cap are identified as vulnerable plaques because of their potential to rupture and are also known as thin-cap fibroatheromas (TCFA). Other typical features of vulnerability are plaque vascularization, plaque volume, matrix metalloproteinase expression, collagenase activity, and macrophage infiltration of the fibrous cap (Figure 1) [22]. In case of plaque rupture, the contact of lipid material with blood activates the coagulation process, which can determine endoluminal thrombosis with partial or total occlusion of the vessel eliciting an acute coronary syndrome. Total coronary artery occlusion can determine myocardial necrosis and subsequent STEMI or NSTEMI, while partial occlusion can induce ischemia without myocardial necrosis that clinically shows as unstable angina; however, in most cases, the plaque undergoes a healing process and remains clinically silent [23,24]. 

Instead, the slow-growing plaques can lead to the development of coronary stenosis of different grades of severity, which can generally exacerbate symptoms during physical exercise, triggering stable coronary artery disease. Plaque size and the degree of remodeling define the percentage of luminal narrowing. Under stress conditions, stenosis between 50% and 90% induce a gradual and proportional reduction of the coronary flow reserve, secondary to a reduced capability of the coronaries to vasodilate and supply oxygen to the myocardial tissue, with consequent myocardial ischemia and thoracic pain [25].

## 3. CCTA Imaging Technique

Coronary imaging with CT remains a challenging task requiring a high spatial resolution and high temporal resolution to provide motion-free images of the coronary arteries [26]. CT scanners could obtain an x–y plane spatial resolution of 0.3 mm even prior to the multislice CT era, but the resolution along the *z*-axis was limited by the need to acquire wider slices to complete the scan in a single breath-hold. The advantage of current CT systems, capable of imaging 64 slices or more, is to achieve an isotropic spatial resolution [27].

To avoid significant image blur, a good temporal resolution (TR) is also needed, which can only be provided by a scanner with a fast gantry rotation speed. To improve TR, specialized cardiac reconstruction algorithms utilizing only 180° of data for image reconstruction can be employed. This enables an intrinsic TR of approximately half the gantry rotation time in a CT scanner with a single X-ray source. On dual-source CT scanners, with the two X-ray tubes located at approximately 90° to each other, sufficient data can be acquired in approximately one-fourth of an entire gantry rotation [27,28].

The length of the heart that must be included in a CTCA scan is generally around 120–140 mm. Since the many high-end CT scanners use a *z*-axis detector length shorter than this, they generally cannot image the whole cardiac volume within a single gantry rotation, but they acquire a series of slabs over several heartbeats. Recently introduced volume CT scanners can acquire the whole cardiac volume (16 cm) in a single heartbeat, consequently avoiding misregistration artefacts, particularly in patients with arrhythmias, reducing the volume of the iodine-based contrast agent and furthermore these scanners have the capability of performing dynamic myocardial perfusion studies [29]. Beyond impressive coronary arterial imaging, these scanners significantly reduce the acquisition time to less than 1 s, also decreasing the radiation dose to the patient. Volumetric scan also eliminates stair-step artifacts, and it is particularly useful for myocardial function evaluation and perfusion imaging [27].

Another approach to achieve whole volume coverage within a single heartbeat, available on Siemens dual-source scanners, is to perform a prospectively ECG-triggered helical scan at a very high pitch (>3). The high table speed allows the whole cardiac volume to be covered in around 250 ms. In this “Flash” mode, the cardiac volume can be acquired within a single heartbeat, although this mode is generally limited to patients with low heart rates, typically 65 bpm [27].

Cardiac CT scanners have evolved from electron beams, capable of detecting coronary calcification, to multidetector scanners, capable of undertaking contrast-based cardiovascular imaging. The advent of wide detector technology, dual-source X-ray, and high-pitch acquisition platforms have significantly improved temporal resolution, but none of these strategies have improved spatial resolution. In CCTA imaging, the primary determinant of spatial resolution is the voxel size that in current clinical scanners is limited to 0.4 mm^3^. Higher spatial resolution relies on thinner collimation, a small detector size and advanced reconstruction algorithms [30]. Other factors involved in the assessment of plaque characterization are tube current and voltage, contrast agents and imaging processing reconstruction. Dual-energy CCTA provides additional data and could improve the differentiation of plaque components. It has been shown that using dual energy-CCTA at 80 and 140 kV, the differentiation between calcified and non-calcified plaques has improved [31]. 

An overview of technical specifications from the latest available CT scanners by different manufacturers has shown in Table 1.

Contrast material affects plaque imaging in relation to its volume, injection rate, iodine concentration, cardiac output and heart rate [32]. Among imaging processing factors, iterative reconstruction significantly lowers noise compared to filtered back projection. This is very important in the case of a thin-cut acquisition to mitigate the increased noise; furthermore, it has been shown that the reconstruction kernel considerably influences plaque characterization. Indeed, a soft reconstruction kernel enhances visual detectability of low-density plaques while the opposite effect may be observed in the evaluation of small calcification [33,34].

At present, 64-detector row CT is considered the minimum standard for CCTA. Image quality improves substantially when the heart rate is regular and lower than 65 beats/minute (b/m), so patients’ preparation is another crucial element. In the absence of contraindication, the administration of beta-blockers and nitrates to lower the heart rhythm and vasodilate coronary arteries is recommended to improve image quality and coronary evaluation. However, the latest CT scanners enable cardiac acquisitions at high frequencies, ensuring sufficient image quality without the need for beta blockers. Moreover, these state-of-the-art CT scanners offer enhanced diagnostic quality, even in cases of heart rate irregularities, such as patients with atrial fibrillation, with the capability of acquiring images in a single beat.

Different acquisition strategies are available to obtain the best image quality based on the patient’s heart rate, BMI and ability to breath-hold. 

## 4. ECG Gating 

**Figure 2:**
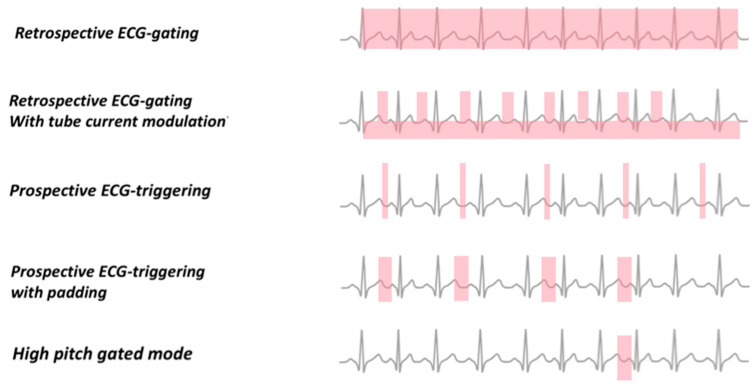
ECG-gating approaches for CCTA.

ECG gating is mandatory to perform cardiac CCTA because all images must be frozen in defined points of the cardiac cycle. Three approaches for ECG gating are mainly used: 1. Retrospective ECG gating with spiral data acquisition. 2. Prospective ECG gating with a sequential data acquisition. 3. Prospective ECG gating with spiral data acquisition and high pitch (Figure 2).

### 4.1. Retrospective ECG Gating 

Using the retrospective ECG-gated spiral scanning technique with multidetector scanners grants high-speed acquisition of the entire heart with a submillimeter spatial resolution and without motion artefacts during a single breath hold.

In this approach, CCTA projections are constantly acquired in the spiral mode, and the ECG signal is recorded at the same time; afterwards, software algorithms can extrapolate the data from different phases of the cardiac cycle by progressively shifting the temporal window relative to the R wave. Through retrospective gating, every position of the heart is acquired by a detector row at every point of the cardiac cycle while the table moves continuously but advances no more than the total width of active detectors for each heartbeat [35]. For this reason, using a 4 cm detector, the coverage of the entire heart volume takes four beats [30,36]. This approach requires a low table feed (pitch < 1) to ensure coverage of the whole heart with a constant tube current and to allow the selection of the optimal reconstruction window throughout the cardiac phase. However, the acquisition of multiple phases is often avoidable in patients with a low and regular heart rate, in which diastole is generally sufficient for optimal reconstruction of coronary arteries. 

In the retrospective ECG-gated spiral scanning technique, the radiologist can use the ECG-controlled tube current modulation to increase tube current during a pre-determined cardiac phase and drastically lower the current for all the other phases that are not considered useful for the diagnosis. Nowadays, ECG-controlled tube current modulation represents one of the most useful CCTA techniques, since it grants an optimal imaging quality reducing the effective dose by approximately 30–50% and consequently the lifetime risk of developing cancer in all patients, particularly for women and younger patients [37,38,39]. In terms of dose reduction, benefits are particularly evident in patients with a low and regular heart rate, which have a full dose window duration shorter than those with a higher and variable heart rate [38]. In CT, the radiation dose is proportional to the square of the tube voltage and proportional to the tube current. Therefore, diminishing the tube voltage lowers the radiation dose far better than reducing the tube current. Lowering the tube voltage from 120 kVp to 100 or 80 kVp can significantly limit radiation dose without losing image quality in children and patients with BMI < 25 kg/m^2^ [40,41,42]. Because of a greater photoelectric effect, diminishing the tube voltage also increases the attenuation of iodinated contrast media which can also grant a smaller volume of contrast medium without lowering attenuation within vessels [38,43].

### 4.2. Sequential Mode Prospective ECG Gating

In the prospective ECG-gated axial scan, or step-and-shoot scan, CT scanning is limited to a defined point of the cardiac cycle, usually during diastole, depending on the patient’s heart rate. Since axial scanning is used, the table is not moving during but only between data acquisition. The X-ray beam activates on the preselected cardiac phase to obtain the necessary data to reconstruct images during the minimal acquisition window, which corresponds to the time for the gantry to rotate 180° plus the size of a fan angle. The total number of heartbeats and number of image stacks for a cardiac CT depends on the width of the detector; in most adult hearts, a scanning of about 12 cm in the *z*-axis is required to cover the total heart volume. Currently, prospective ECG gating is the most widely used technique for data acquisition in cardiac CT since it is associated with an average radiation dose of 2.7 mSv [44,45,46]. Compared with the retrospective ECG-gated helical scan, this technique lowers the radiation dose essentially by reducing the acquired cardiac phases and by increasing the pitch to 1, with minimum overlapping between the scans. Furthermore, this approach can also limit blurring in the coronary arteries, particularly if calcification or stents are present, and can enhance image quality. For follow-up after bypass graft surgery, which needs a wider scan coverage to include the grafts and the native coronaries, the step-and-shoot scan could represent a reasonable choice to reduce the effective dose in these patients [47]. 

However, this protocol is generally limited to patients with a regular heart rate inferior to 70 beats per minute and, since the scan is sequentially obtained, there is no or minimal flexibility in retrospectively choosing different phases of the cardiac cycle for image reconstruction. The padding technique can “enlarge” the acquisition window, adding extra tube-on time before and after the pre-determined window to obtain additional data during different cardiac phases; against, padding obviously increases radiation dose (45% increase per 100 ms increase in padding time) but also improves image quality for patients with low heart rate variability [48].

### 4.3. Prospective ECG Gating with Spiral Data Acquisition and High Pitch

A dual-source CT scanner can accomplish gapless z-sampling with a pitch as high as 3.4 to obtain full coverage of the heart volume during a single cardiac cycle in a scanning time of 250–290 ms, providing a “snapshot” of the entire heart, usually during diastole. The table moves at a very high speed to cover the total heart volume within a single beat. A quarter rotation of data per measurement system is used for image reconstruction, and each of the individual images has a temporal resolution of a quarter of the gantry rotation time. A prerequisite for this approach is a stable sinus rhythm with a heart rate ≤ 60 b/min, to reduce motion artefacts. The effective radiation dose of this approach can be below 1 mSv [38,49,50,51,52]. When applying this technique, the table continuously moves during scanning and with prospective triggering by the patient’s ECG, one X-ray tube rotates around the patient without overlap at a pitch as high as 3.4, while the other tube rotates a quarter rotation later at the same pitch to fill the sampling gaps [50,53]. Afterwards, images are reconstructed with a temporal resolution of 75 ms (half-scan reconstruction), and subsequent images are reconstructed at progressively later times within the cardiac cycle, so that the obtained dataset is not uniform in time, with the most proximal scans in end-systole or early diastole and with the most distal ones in later diastole [50,53]. In conclusion, when a dual-source CT scanner is available, a prospective ECG-gated high-pitch acquisition mode could represent an option to capture a single phase of the cardiac cycle, with the lowest possible dose in cooperative patients with a stable sinus rhythm lower than 60 bpm and a maximum patient weight of 100 kgs. 

**Figure 3:**
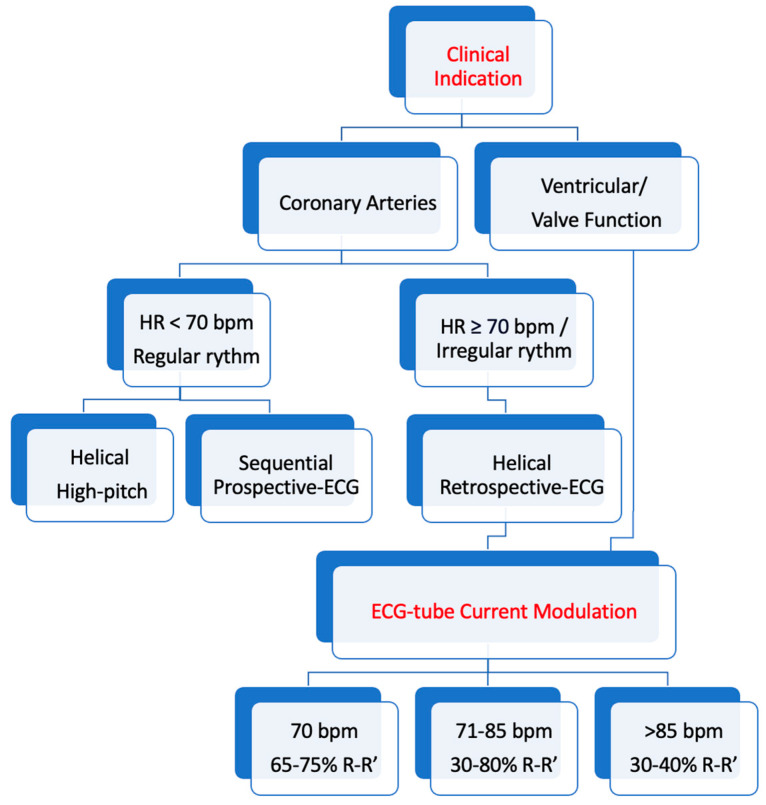
Flowchart proposal for CCTA protocol.

Choosing the right scanning mode depends on the patient’s heart rate. A heart rate below 70 beats per minute would be desirable: in this type of patient, it is possible to choose between a high-pitch helical acquisition mode or a prospective sequential scan. In patients with a heart rate greater than 70 beats per minute or with an irregular rhythm, it is mandatory to use a retrospective helical scan mode (Figure 3). However, it is important to consider that in patients uncompliant to breathing commands, the images obtained may be degraded by motion artifacts. When dealing with patients experiencing extrasystole, it is essential to be aware of the different acquisition modes. In sequential acquisition, the extrasystole is deliberately omitted from the recording. On the other hand, in retrospective mode, the extrasystole is included in the acquisition and can be later edited out during post-processing.

With the advent of last generation scanners, prospective ECG gating with systolic triggering (45% R–R interval, plus 100 milliseconds of padding if necessary) could represent an invaluable resource in challenging patients affected by atrial fibrillation (AF). The technique allows coronary arteries evaluation with high image quality and without an increase in radiation exposure in AF patients, even with a high heart rate. Prospective gating with systolic acquisition offers image quality which is at least comparable to retrospectively gated studies, improving diagnostic confidence at a significantly reduced radiation dose [54].

Regarding radiation dose, the introduction of iterative reconstructions has favored the development of low kV protocols. Prospective ECG-gating can remarkably reduce the radiation dose but using low kV scanning will further decrease radiation dosage and enhance vascular CT value, allowing a lower dose of contrast agent but with a higher noise-for-image quality. A low-concentration contrast agent reduces the intake of iodine and possibly decreases its vascular concentration. Iterative reconstruction can increase image quality by reducing noise. A low kV and low concentration of contrast agent combined with iterative reconstruction for CTCA imaging produced an image quality consistent with that of conventional CTCA and significantly reduced the dosage of the radiation and injected iodine [55]. Furthermore, the recent advancements in artificial intelligence (AI) have led to the development of different deep-learning image reconstruction algorithms which offers unique opportunities for reducing image noise without degrading image quality or diagnostic accuracy in CCTA. These algorithms can provide a reduction in radiation dose from CCTA even of 43% without significant impact on image noise, stenosis severity, plaque composition, and quantitative plaque volume [56].

## 5. Coronary Artery Calcium Scoring

First developed in the early 1990s, the Agatston coronary artery calcium (CAC) score is an international guideline-endorsed decision aid for risk assessment and personalized management in the primary prevention of CAD [57]. With current available CT scanners, even non-gated studies can allow either semiquantitative or quantitative CAC. Generally, CAC is evaluated via a standardized protocol using prospective ECG-triggered axial scanning at 3 mm with 120 kV tube voltage. The calcium scale comprises four categories: normal (0), mild (1–99), moderate (100–400) and severe (>400). Nowadays, calcium score can be obtained at about 1 mSy of radiation, without the need for contrast agents. CAC has been proven valuable in the management of CAD and primary prevention, while the benefit of moderate values of CAC to predict prognosis is still controversial [57].

## 6. High-Risk Plaque Features at CCTA 

An overview of the main studies assessing high-risk plaque features with CCTA is shown in Table 2.

### 6.1. Low Attenuation

**Figure 4:**
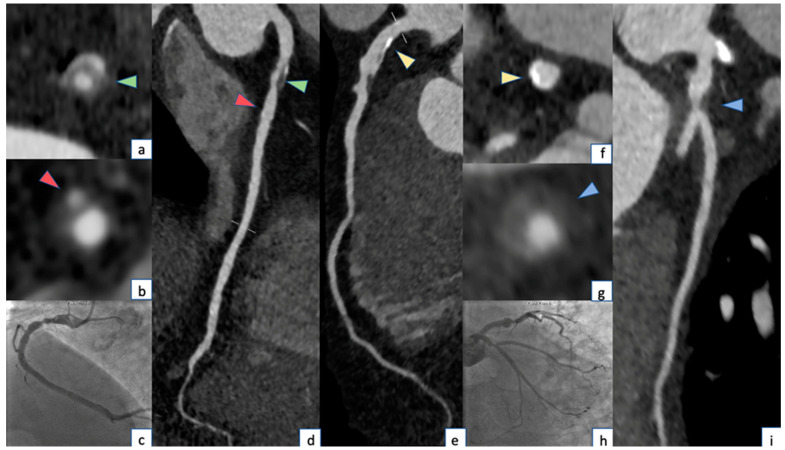
CCTA and coronary angiography of a 74-year-old male patient showing: Two plaques in the proximal segment of RCA with respective napkin-ring sign (green arrowhead, (**a**–**d**)) causing significant stenosis (>70%) and spotty calcification (red arrowhead, (**b**–**d**)) producing mild stenosis. Calcified plaque in the proximal segment of LAD causing mild stenosis (yellow arrowhead (**e**,**f**)). Positive remodeling and low attenuation plaque in the proximal segment of CX (blue arrowhead, (**g**–**i**)). The corresponding coronary angiography images are shown in figure (**c**–**h**).

CCTA characterization of atherosclerotic plaques relies on morphological features and on the correct identification of the biochemical composition. The Hounsfield Units (HU) values for the various plaque constituents range from −30 to 60 for lipid plaques, from 61 to 149 for fibrous plaques and from 150 to 1300 for calcium [58,59] (Figure 4). However, the CT attenuation value of lipid plaques is similar to that of fibrous plaques, so it is complex to characterize plaques by CCTA attenuation alone. Furthermore, the CCTA value of plaques is influenced by various factors such as slice thickness, tube voltage and contrast agent. CCTA can be a valuable tool to quantify the burden of calcified, non-calcified and low-attenuation plaques, as well as the total coronary plaque burden, providing important prognostic information. In a recent study, Williams et al. stated that low-attenuation plaques represent the strongest predictor of fatal or non-fatal myocardial infarction, surpassing other well-known cardiovascular risk markers such as score systems, computed tomography calcium scoring and coronary artery stenoses. The authors discovered that patients with a low-attenuation plaque burden > 4% were five times more likely to suffer myocardial infarction [60].

Moreover, Deseive et al. investigated the prognostic value of low-attenuation plaque volume (LAPV) from the CCTA datasets of 1577 patients with suspected CAD that were followed for 5.5 years using death and ACS as the primary endpoints. They found that quantified LAPV provided incremental prognostic information beyond clinical risk, obstructive CAD and CACS. A combined approach using quantified LAPV and clinical risk may offer cumulative prognostic information beyond well-known CT risk patterns and could be helpful for the stratification of patients into low, intermediate and high-risk categories compared to clinical risk [61].

Furthermore, in a recent retrospective study, Yamaura et al. evaluated the prognostic impact of low-attenuation non-calcified coronary plaque volume and its association with epicardial adipose tissue (EAT) volume in 376 patients without known CAD. They determined the percentage LAP volume (%LAP, <30 HU) as the LAP volume divided by the vessel volume, while EAT was defined as fat tissue with a CT attenuation value between −250 and −30 HU. The main aim of the study was to understand how the combination of these two factors impacted their primary endpoint, described as an event of death, non-fatal myocardial infarction, unstable angina or worsening symptoms which required unplanned coronary revascularization. The authors discovered that %LAP was an independent predictor of the primary endpoint (hazard ratio [HR], 3.05) and that CACS and EAV can be useful in determining %LAP. These three factors could help improve personalized cardiac risk management by administering patient-suited therapy [62].

### 6.2. Positive Remodeling

Atherosclerotic plaque initially tends to grow outwards, leaving the luminal integrity unchanged [63] (Figure 2). Therefore, many coronary plaques accumulate lipids and become more complex without causing any clinical symptoms. Autoptic studies on sudden cardiac death cases found that plaques with positive remodeling have larger lipid cores and more macrophages, which are markers of vulnerable plaques [64]. The remodeling index (RI), using CCTA, is expressed by the ratio between vessel cross-sectional area at the level of maximal stenosis divided by the average of the proximal and distal reference sites [65]. Given this, RI > 1.1 is considered for positive remodeling [66]. It has been shown that plaques with positive remodeling are more frequent in patients with acute coronary syndrome (ACS) compared to those with stable angina [67]. Positive remodeling has the best sensitivity and specificity for identifying patients with ACS if compared with low attenuation and spotty calcifications [68]. Positive remodeling is less dependent on image noise than plaque attenuation and has a more quantitative definition. For these reasons, it might become a more robust marker to detect vulnerable plaques. However, more studies are required to assess the effect of positive remodeling on later outcomes. In a recent SCOT-HEART analysis, Williams et al. investigated the prognostic implications of adverse coronary plaque characteristics, such as positive remodeling and low attenuation in patients with suspected CAD. They found that major adverse cardiac events were three times more frequent in patients with high-risk feature plaques. Positive remodeling was the most frequent among all the features, followed by spotty calcifications and low attenuation, which demonstrates that these features confer an increased risk of CAD-related death or non-fatal myocardial infarction [69].

### 6.3. Spotty Calcifications

Several histological studies have shown calcified nodules in patients with coronary thrombosis and in sudden death cases [70,71,72]. On this basis, some authors speculate that intra-plaque microcalcification might promote plaque rupture [73]. Spotty calcifications are defined as <3 mm calcified plaque elements with a 130 HU attenuation value, surrounded by non-calcified plaque tissue and have been proposed as a CCTA marker of histological microcalcification [67,74] (Figure 2). However, only calcifications greater than 0.5 mm in diameter are visible on CCTA, so most of them cannot be recognized since the current resolution of clinical scanners is under the threshold needed for identifying microcalcifications [75,76].

Van Velzen et al. were among the first to compare calcification patterns in plaques on CCTA with plaque characteristics on intravascular ultrasound with radiofrequency backscatter analysis (IVUS-VH). They identified three different calcification plaque patterns: non-calcified, spotty or dense calcifications. Moreover, spotty calcifications were distinguished into small (<1 mm), intermediate (1–3 mm) and large (≥3 mm) spotty calcifications. At IVUS-VH, the two main high-risk characteristics are the percentage of a necrotic core (NC) and the presence of a thin cap fibroatheroma (TCFA). At CCTA, they found that plaques with small spotty calcifications had higher %NC and %TCFA, suggesting that CCTA may be an exciting tool to assess vulnerable plaques [77]. However, the literature is still poor concerning the prognostic value of spotty calcification at CCTA since they are better evaluated through OCT with higher resolution (10–20 μm) compared with non-invasive CT [78].

In the ROMICAT II trial, spotty calcium was the most common high-risk plaque feature, being present in 151 patients [79]. Notably, the authors revealed that the detection of high-risk coronary plaque on CCTA among patients presenting with acute chest pain was significantly associated with ACS, regardless of the presence of significant CAD and clinical risk assessment.

### 6.4. Napkin-Ring Sign

The napkin-ring sign (NRS) can be evaluated on the cross-section of non-calcified plaques and exhibits two main features: a low-attenuation central area, surrounded by annular high attenuation tissue [10,80] (Figure 2). The first one corresponds to the large necrotic core while the annular area is considered fibrous tissue. Ring density is greater than that of the inner core but <130 HU in the CCTA scan. At present, this sign is considered a reliable marker of plaque instability [10,23]. There are many controversies regarding the predictive value of different CCTA high-risk plaque features to foresee future acute coronary events [81,82,83]. However, an analysis of the SCOT HEART study stated that patients with one or more adverse plaque features, especially in the presence of a stenosis, had a three-time higher risk of myocardial infarction [69]. To establish which of the feature has the most significant value is not an easy task. 

Otsuka et al. evaluated the predictive value of the napkin-ring sign on CCTA in a group of 895 patients with CAD that were followed up for 1 to 3 years for ACS events (sudden cardiac death, myocardial infarction or unstable angina). Their statistical analysis showed that positive remodeling (*p* < 0.001), low attenuation plaque (*p* = 0.007) and the napkin-ring sign (*p* < 0.0001) were all independent predictive factors for future ACS events and that NRS presented a higher risk of MACE compared to the other features, highlighting that NRS represent a solid independent predictive factor for MACE in CAD [80].

Recently, Feuchtner et al. tried to assess the prognostic value of CCTA for predicting MACE over a long-term follow-up period in patients with CAD [84]. Their statistical analysis revealed that LAP < 60 HU and NRS were the strongest MACE predictors with a hazard ratio of 4.96 (95% CI: 2.0–12.2) and 3.85 (95% CI: 1.7–8.6), respectively, while the other features were less powerful, demonstrating that NRS, together with low attenuation, has the most reliable predictive value of MACE.

### 6.5. Pericoronary Fat

Pericoronary fat is a particular type of adipose tissue. It can interact with adjacent coronary walls through a paracrine manner, changing its phenotype in response to signal from the arterial layers [85,86,87]. Inflamed blood vessels can release biochemical signals that directly reach the epicardial pericoronary fat, which can stimulate local lipolysis and enhance microvascular permeability promoting perivascular oedema; these changes determine different gradients of adipocytes around the vessel. On this basis, the attenuation of pericoronary fat measured by CCTA has been revealed as an indicator of high-risk plaques. It has been shown that culprit lesions in ACS were associated with increased the attenuation of pericoronary fat around the lesion [88,89]. However, the attenuation of pericoronary fat could also be affected by other factors such as angiogenesis, inflammation and fibrosis [90]. The pericoronary fat attenuation index (FAI) on CCTA has been suggested as a new marker of coronary vascular inflammation with a prognostic value for MACEs.

Sun et al. used CCTA to evaluate the pericoronary fat attenuation index (FAI) as a novel imaging biomarker of coronary inflammation. The main aim was to assess whether increased pericoronary FAI values were associated with vulnerable plaque features in patients with non-ST elevation ACS. The authors evaluated 195 lesions in 130 patients with non-ST elevation ACS. Lesion-specific pericoronary FAI, plaque components and other plaque features were assessed by CCTA. The group found that plaques with FAI values >70.1 HU exposed spotty calcification and low attenuation more frequently than plaques with lower FAI values; moreover, they were associated with an increased proinflammatory intracellular cytokine profile. These results suggest that a pericoronary FAI value >70 HU could be considered a marker of local immune-inflammatory response activation strongly related to plaque vulnerability [91].

**Table 2:** Main studies assessing high-risk plaque features with CCTA.

First Author	Publication Year	Study Design	Patients (n)	Aim of the Study
Williams [60]	2020	Multicenter randomized controlled trial	1769	To explore whether the quantification of low-attenuation plaque identified by CCTA enhances the ability to predict fatal or nonfatal MI when compared to traditional cardiovascular risk scores, Agatston scoring and the severity of obstructive CAD in stable patients presenting with chest pain.
Williams [69]	2019	Multicenter randomized controlled trial	4146	To assess the prognostic implications of adverse coronary plaque characteristics with CCTA.
Puchner [79]	2014	Multicenter randomized controlled trial	1000	To assess whether the identification of high-risk plaque features detected by CCTA in the emergency department can enhance the diagnostic accuracy of ACS beyond the presence of significant CAD and clinical risk assessment in patients experiencing acute chest pain but without objective evidence of myocardial ischemia or MI.
Otsuka [80]	2013	Prospective study	960	To determine the predictive value of the napkin-ring sign detected by CCTA for future ACS events in patients with CAD.
Sun [91]	2022	Prospective study	130	To examine the correlation between pericoronary inflammation and plaque morphology and components with CCTA in individuals with non-ST elevation ACS

MI: myocardial infarction; ACS: acute coronary syndrome; CAD: coronary artery disease.

Similarly, in the Cardiovascular RISk Prediction using Computed Tomography (CRISP-CT) study, Oikonomou et al. analyzed outcome data obtained prospectively from two groups of patients undergoing CCTA. They evaluated the prognostic value of FAI for all-cause and cardiac mortality and discovered that perivascular FAI improves patient cardiac risk prediction and stratification, offering a quantitative measure of coronary inflammation. High perivascular FAI values (≥70.1 HU) indicate increased cardiac mortality and could guide early targeted primary prevention in patients [92].

Finally, in a recent meta-analysis, Sagris et al. evaluated twenty studies to assess differences in FAI values between stable and unstable coronary plaques and establish the hazard ratio (HR) of high FAI values to predict future cardiovascular risk events. From their meta-analysis, it emerged that FAI values are higher in unstable than stable plaques with a mean difference of 4.50 HU [95% confidence interval (CI): 1.10–7.89, I = 88%. Furthermore, in prospective follow-up studies (6335 patients), higher pericoronary FAI values offered incremental prognostic values for MACEs (HR = 3.29, 95% CI: 1.88–5.76). In conclusion, pericoronary FAI could be considered a promising imaging marker for the detection of coronary inflammation to discern between stable and unstable plaques, and for the prediction of future MACEs [93].

### 6.6. Geometry of Coronary Plaques

Several authors have explored the relationship between the geometric characteristics of plaques, the involved segments, and their impact on the development of atherosclerosis and the occurrence of MACE [94,95]. A wealth of 2D and 3D studies has identified various geometric parameters that contribute to this understanding. These parameters include but are not limited to the length of stenosis, the volume of lumen in the stenosed segment as well as that of calcified and non-calcified components, the cross-section area of non-calcified components, and the tortuosity of the lesion and of coronary arteries [95]. Interestingly, in their case-control study the authors showed that among the 548 coronary lesions observed in 116 patients who experienced incident ACS after CCTA, the lesions situated in close proximity to the ostium or within vessel bifurcations or tortuous segments were more prone to evolving into culprit lesions. The assessment of plaque severity is commonly gauged through diameter and area ratios. Certain secondary geometric parameters, derived from the 3D extraction of coronary plaques, may hold associations with the specific location of plaque rupture and subsequent clinical events [94]. 

## 7. Future Perspectives and Conclusions

Cardiac imaging has always represented a challenging technique due to cardiac and respiratory motion. Recent technological developments, such as advanced multi-detector row scanners, improved gantry rotation times and acquisition and post-processing software development, pushed to the limit cardiac single energy CT (SECT) scans first, dual source and dual energy CT (DECT) then, and now have unlocked photon-counting CT (PCCT) [96].

As discussed above, CCTA can identify many high-risk plaque features, such as low attenuation, positive remodeling, spotty calcifications and the napkin-ring sign.

In this setting, DECT could be of value in evaluating vulnerable plaques thanks to its capability to use X-rays at different energies, which affect the attenuation values of different plaque components, such as fibrous tissue and necrotic core. However, the literature on plaque evaluation by DECT is scarce and contradictory [97]. DECT can discern calcified from non-calcified plaques without gaining a real advantage over SECT for classifying plaque subtypes. Nevertheless, Obaid et al. recently demonstrated that the use of CCTA at two separate energy levels (100 and 140 kV) can enhance the sensitivity and specificity for recognizing the necrotic core of plaques ex vivo, while the in vivo diagnostic accuracy for the identification of necrotic core is still suboptimal [98]. Furthermore, Tanami et al. stated that at lower energy settings (80 kV), CT analysis of ex vivo plaques could ensure better results in distinguishing lipid-rich plaques from fibrotic plaques [99]. Moreover, the authors proposed that the ratio of the CT attenuation value at 80 kV divided by the 140 kV value (Hounsfield ratio [HR], 80:140) could represent a practical tool for plaque classification [99]. 

Calcium and stent blooming still represent a challenge. Blooming artefacts are recognized for exaggerating the actual volume of both calcium plaques and stent meshes. Consequently, radiologists may encounter a tendency to overestimate the grade of stenosis [100]. These artefacts are related to the partial volume effect, motion artefacts and beam hardening. In contemporary times, the influence of beam hardening has significantly diminished with the advent of advanced CT scanners. Possible solutions, therefore, hinge on advancements in high-resolution CT hardware and reconstruction methodologies, including two-pass subtraction techniques and sophisticated post-processing methods [100]. In particular, CT vendors are advancing the field with the development of cutting-edge photon-counting technologies that boast significantly enhanced spatial resolution capabilities compared to the majority of available scanners. This innovation is specifically targeted at mitigating the issue of calcium blooming. Another approach to address blooming involves the utilization of advanced high-resolution CT reconstruction methods. Subtraction techniques aim to distinguish dense elements from the vessel lumen by leveraging dual-energy data. Through subsequent material decomposition, these techniques measure the volume fraction of the calcified component in each voxel, allowing for its subtraction. Alternatively, a dual-contrast approach envisions employing two scans with varying contrast levels. The subtraction of these scans proves beneficial in isolating the lumen, resulting in a calcium or stent-free image that facilitates easier diagnosis. Post-processing techniques, such as deconvolution or DL algorithms, exhibit considerable promise in effectively correcting blooming artifacts [100]. 

In this setting, the recently introduced PCCT could represent a game-changer in cardiac imaging and plaque characterization. PCCT can count the number of X-ray photons and their energy distribution, increasing the contrast-to-noise ratios and the energy-discrimination capabilities. Thanks to its increased spatial resolution, PCCT can also be helpful to depict high-risk plaque features, such as thin-cap fibroatheroma or microcalcifications, raising the CCTA predictive value [101]. Holmes et al. recently investigated the image quality of a dual-source photon-counting CT scanner’s ultra-high-resolution (UHR) mode in evaluating mixed (soft and hard) coronary artery plaques on a custom-made phantom with ten mixed plaques of various sizes and compositions. They discovered that UHR PCCT improves plaque characterization through enhanced spatial resolution, lowering blooming artefacts and partial volume effects [102]. 

In a similar study, Rajagopal et al. compared the performance of an energy-integrating detector (EID) CT, PCCT and high-resolution PCCT (HR-PCCT) for the evaluation of plaques and the reduction of stent artefacts using a phantom. Their results stated that, despite the increased noise, HR-PCCT images could visualize coronary plaques better and reduce stent artefacts compared with EID or PCCT [103].

Recently, Si-Mohamed et al. utilized ex vivo histologic analysis to evaluate the uptake of gold nanoparticles by macrophages in plaques and to compare macrophage counts with the measured concentrations in vivo. The uptake of gold nanoparticles was detectable using PCCT, and they could easily be differentiated from the calcifications and iodine. Histologic analysis showed a clear linear relationship between the concentrations of nanoparticles in the plaques and the concentration measured in vivo. The authors demonstrated that increased amounts of gold nanoparticles at PCCT are firmly related to higher numbers of macrophages in atherosclerotic plaques. This was the first paper that shed light on the invaluable potential of PCCT in molecular atherosclerotic plaque characterization, which can lead to a more accurate depiction of atherosclerotic plaque components adding essential prognostic information beyond the degree of luminal narrowing [104,105]. 

Additionally, computational fluid dynamics (CFD) has been used with promising results to simulate the hemodynamics around plaques [106].

Finally, radiomics represent an emerging research field aimed at gaining diagnosis, characterization and prognosis of diseases by the automatic or semi-automatic quantitative analysis of standard medical images [107]. Although radiomics is mainly applied to oncologic research to extract information concerning tumor features [108], there is an increasing interest in its usage in cardiac imaging [109,110] and many authors are wondering if radiomics could play a role in the characterization of vulnerable plaques [111].

Since visual and histogram-based assessments of CCTA angiography have limited accuracy in the identification of vulnerable plaques, Kolossvary et al. developed a radiomics-based machine learning (ML) model to evaluate its diagnostic performance in advanced atherosclerotic lesions. In their prospective study, the group imaged 21 coronary arteries from seven hearts ex vivo with CCTA. Of 95 coronary plaques, they coregistered 611 histologic cross-sections with CCTA cross-sections. Early fibroatheroma, late fibroatheroma or thin-cap atheroma were considered advanced plaques. Upon visual evaluation, CCTA lesions were divided into homogeneous, heterogeneous or napkin-ring sign plaques. The authors also considered the low attenuation (<30 HU) and the average HU of the plaques. Among eight radiomics-based ML models trained on the training set (75% of the cross-sections), the best-performing one was compared to the visual assessment and histogram-based evaluation on the validation set (25% of the cross-sections). The selected radiomics-based ML model outperformed visual assessment (AUC = 0.73 vs. 0.65), area of low attenuation (AUC = 0.55) and average HU (AUC = 0.53) in the detection of advanced atheromatous plaques, suggesting that radiomics-based ML analysis increases the capability of CCTA in characterizing atherosclerotic plaques [112]. 

Similarly, Li et al. explored radiomics-based ML model performances in detecting vulnerable plaques at CCTA. The researchers utilized pathological cross-section samples of 350 plaques from 36 end-stage hearts collected and coregistered them to patients’ preoperative CCTA images. Afterwards, the authors derived eight radiomics-based ML models for lesion vulnerability prediction and tested them on an independent set of 196 plaques from another group of patients. In the validation group, diagnosis based on CCTA parameters demonstrated moderate ability (AUC: 0.656 [95% CI: 0.593–0.718]), while the radiomics model showed better diagnostic performances (0.782 [95% CI: 0.710–0.846]), suggesting that radiomics models could reach a better diagnostic accuracy than conventional CCTA features at assessing plaque vulnerability [113].

In a recently published paper, Chen et al. evaluated the performance of CCTA-based radiomic signature of vulnerable plaques defined with intravascular US to predict an increased risk for MACE. The signature of vulnerable plaques was developed through a data set including patients undergoing CCTA and then intravascular US. Afterwards, the authors evaluated the prognostic value of the radiomics signature for predicting MACE on a prospective cohort with suspected CAD. After an accurate analysis, sixteen radiomic features were chosen to build the signature, which gained a moderate-to-good AUC in the training, validation, internal and external test sets (AUC = 0.81, 0.75, 0.80, and 0.77, respectively). A high radiomic signature (≥1.07) was independently associated with MACE over a median 3-year follow-up (hazard ratio, 2.01; *p* = 0.005), suggesting it could represent a valuable tool for the detection of vulnerable plaques with increased risk for MACE [114]. Many ML and DL algorithms have been proposed for the automatic detection and classification of coronary plaques. These algorithms can improve clinical workflow efficiency, enhancing radiologists’ performance, improving early diagnosis and risk stratification and increasing timeliness of image interpretation [115]. In addition, CCTA cannot completely replace other invasive imaging techniques such as IVUS or OCT to detect essential plaque characteristics such as erosion and neovascularization. There are few studies that have explored the feasibility and development of ML or DL algorithms capable of collecting, comparing and merging data from various modalities to characterize vulnerable plaques [115]. AI algorithms using ML- and DL-based methods have merits for identifying plaques, and can be used as a valuable resource in the medical decision-making process. 

In conclusion, CCTA represents a robust imaging technique to noninvasively detect, quantify and characterize coronary atherosclerotic plaque, but technical optimization and image quality are mandatory. Imaging features related to high-risk plaques include low attenuation, positive remodeling, spotty calcification, napkin ring and high FAI, with several prognostic data provided with CCTA. The non-invasive detection of high-risk plaque features via CCTA can improve the risk assessment for MACE, particularly in patients with non-obstructive CAD, younger patients and women. CCTA high-risk plaque features may represent a pivotal element for risk stratification and an invaluable tool for clinical management and personalized therapy [92]. 

Promising future perspectives for plaque characterization are expected with the introduction of PCCT and the implementation of radiomics and AI approaches.

## Figures and Tables

**Table 1:** Overview of technical specifications from the latest available CT scanners.

	Revolution Apex (GE)	IQon Spectral CT (Philips)	Somatom Definition Flash (Siemens)	Somatom Definition Drive (Siemens)	Somatom Definition Force (Siemens)	Naeotom Alpha (Siemens)	Aquilion One (Canon)
**Detector type**	Gemstone scintillator	Spectral Detector –NanoPanel Prism	2× Multislice Stellar detector	2× Multi-slice StellarInfnity	2× Multislice StellarInfinity	2× QuantaMax	2× Pure Vision
**Detector rows**	256	64 rows, 256 slice	128 (2 × 64)	128 (2 × 64)	384 (2 × 192)	288 (2 × 144) or 240 (2 × 120)	640
**Gantry aperture, cm**	80	70	78	78	78	82	78
***z*-axis,** **mm**	160	40	38.4	38.4	57.6	60	160
**Rotation time, sec**	0.23	0.27	0.28	0.28	0.25	0.25	0.27

## Data Availability

Not applicable.
